# Draft genome sequence of *Marinimicrobium* sp. ARAG 43.8

**DOI:** 10.1128/mra.00470-25

**Published:** 2025-08-20

**Authors:** Trinh Thi Van Anh, Nguyen Bao Chau, Vu Nguyen Thanh, Nguyen Thanh Thuy

**Affiliations:** 1 Food Industries Research Institute, Hanoi, Vietnam; California State University San Marcos, San Marcos, California, USA

**Keywords:** agar, genome, *Marinimicrobium*

## Abstract

The draft genome sequence of *Marinimicrobium* sp. ARAG 43.8, an agarolytic bacterium isolated from dewatered sewage sludge of an agar factory in Hai Phong, Vietnam, is presented here. The genome is 3,667,906 bp in length, with a G + C content of 56.58%.

## ANNOUNCEMENT

The genus *Marinimicrobium* was first described in 2006 and comprises five species (www.bacterio.net) (1). To date, 14 genomes representing 4 species are available in the NCBI database. In this study, strain ARAG 43.8 was isolated from dewatered sewage sludge at the Vietxo agar factory in Hai Phong, Vietnam (20°49′1.74″N, 106°36′28.44″E). A total of 12 samples were collected from different areas of the factory, including the raw material warehouse, production line, waste materials, and sewage sludge. Agarolytic bacteria were enriched by incubating approximately 1 g of each sample in 50 mL of M9 broth containing 0.1% agar for 24 hours at 30°C, with shaking at 150 rpm. The suspensions were streaked onto M9 agar plates and incubated at 30 °C for 48 hours. Colonies forming visible depressions in the medium, indicating agarolytic activity, were selected and maintained on Marine Agar 2216 medium (Himedia) (2). In total, 45 strains were obtained. Genomic DNA of strain ARAG 43.8 was extracted and purified using the GeneJET Genomic DNA Purification Kit (Thermoscientific). The 16S rRNA gene was amplified and sequenced using the universal primers 8F and 1492R (3). The obtained sequence (GenBank number PV235060) showed the highest similarity (97.5%) to *Marinimicrobium koreense* DSM 16974^T^ (RJUK01000005) based on comparison against the EzBioCloud 16S rRNA gene database (https://www.ezbiocloud.net).

The genome of ARAG 43.8 was prepared for sequencing using the NEBNext Ultra DNA Library Prep Kit (New England Biolabs, USA) and sequenced on the Novaseq Illumina platform with a 2 × 150 paired-end protocol. A total of 7,907,404 raw reads were generated. The reads were quality-trimmed by Trimmomatic v.0.36 with an average quality score of 30 and subsequently assessed using FastQC v.0.12.1 (4, 5). Genome assembly was performed using SPAdes v.4.1.0 (6). Contigs under 500 bp were filtered out, and the assembled genome was assessed using QUAST v.5.3.0 (7). Draft genome annotation was performed by the NCBI Prokaryotic Genome Annotation Pipeline v.6.10 (8). Carbohydrate-active enzymes were identified using the dbCAN3 (9). The average nucleotide sequence identity between the genome of ARAG 43.8 and *Marinimicrobium* spp. type strains available in the GenBank was calculated using FastANI v.1.33 (10).

All reads were assembled into 36 contigs over 500 bp, with an N_50_ contig length of 443,689 bp and the largest contig of 1,205,655. The draft genome assembly was 3,667,906 bp in length, with a mean G + C content of 56.58% and 323× coverage. Genome annotation revealed 3,146 protein-coding genes, 3 rRNAs, and 37 tRNAs. A total of 16 putative agarolytic genes were identified, including 14 targeting β−1,4-glycosidic linkages (comprising seven GH16, five GH50, and two GH86) and 2 targeting α−1,3-glycosidic linkages (two GH117). The genome sequence showed pairwise average nucleotide identity of 80.03%, 78.65%, 78.57%, and 71.06% with the available genome sequences of *M. agarilyticum* (GCA_000423345.1), *M. koreense* (GCA_003762925.1), *M. locisalis* (GCA_039822625.1), and *M. alkaliphilum* (GCA_003259155.1), respectively. These values are below the established thresholds for bacterial species delineation (11). This result suggests that the strain ARAG 43.8 represents a novel species of the genus *Marinimicrobium*.

## Data Availability

The 16S rRNA gene sequence was deposited in the GenBank under the accession number PV235060. The raw reads and genome assembly of *Marinimicrobium* sp. strain ARAG 43.8 are available in GenBank under accession number SRR33638363 and JBNJLT000000000.1, with associated BioSample SAMN48175420 and BioProject PRJNA1256246. Carbohydrate-active enzyme annotation data generated by the dbCAN3 server are publicly available at https://zenodo.org/records/15730882.
